# Correction: Exercise prescription for the prevention and treatment of chronic diseases in primary care: Protocol of the RedExAP study

**DOI:** 10.1371/journal.pone.0326746

**Published:** 2025-06-18

**Authors:** Alicia Saz-Lara, José Alberto Martínez Hortelano, María Medrano, Raquel Luengo-González, Miriam Garrido Miguel, Montserrat García-Sastre, José Ignacio Recio-Rodriguez, Daniel Cuesta-Lozano, Iván Cavero-Redondo

The eighth author’s name is spelled incorrectly. The correct name is: Daniel Cuesta-Lozano.
