# Supplementary material for: Exercise prescription for the prevention and treatment of chronic diseases in primary care: Protocol of the RedExAP study
Source: PLoS One. 2024 Jul 5;19(7):e0302652. doi: 10.1371/journal.pone.0302652 (PMC11226089; doi:10.1371/journal.pone.0302652)
Supplement: S1 Appendix — (PDF) [file pone.0302652.s001.pdf]

SF-12 HEALTH QUESTIONNAIRE

Instructions:

The questions that follow are about what you think about your health. Your answers will how you feel and to what extent you are able to do your usual activities.

Please answer each question by checking a box. If you are not sure how to answer a question, please answer whichever is more true for you.

1. Overall, you would say your health is:

- o Excellent
- o Very good
- o Good
- o Fair
- o Poor

2. Does your current health limit you to moderate exertion, such as moving a table, vacuuming, bowling, or walking for more than an hour?

- o Yes, it limits me a lot
- o Yes, it does limit me a little bit
- o No, it doesn't limit me at all

3. Does your current health limit you in climbing several flights of stairs?

- o Yes, it limits me a lot
- o Yes, it limits me somewhat
- o No, it does not limit me at all

4. During the last 4 weeks, did you do less than you would have liked to do, because of your physical health?

- o Yes
- o No

5. During the last 4 weeks, did you have to miss any work or daily activities because of your physical health?

- o Yes
- o No

6. During the last 4 weeks, did you do less than you would have liked to do because of any emotional problems (such as being sad, depressed, or nervous)?

- Yes
- No

7. During the last 4 weeks, did you not do your work or daily activities as carefully as usual because of any emotional problems (such as being sad, depressed, or nervous)?

- Yes
- No

8. During the last 4 weeks, to what extent has the pain made your usual work (including work outside the home and housework) difficult?

- Always
- Almost always
- Sometimes
- Many times
- Only some of the time
- Never

9. During the last 4 weeks, how much of the time did you feel calm and collected?

- Always
- Almost always
- Sometimes
- Many times
- Only some of the time
- Never

10. During the last 4 weeks, how much of the time did you have a lot of energy?

- Always
- Almost always
- Sometimes
- Many times
- Only some of the time
- Never

11. During the last 4 weeks, how much of the time did you feel discouraged and sad?

- Always
- Almost always
- Sometimes
- Many times
- Only some of the time
- Never

12. During the past 4 weeks, how often has physical health or emotional problems made it difficult for you to engage in social activities (such as visiting friends or family)?

- Always
- Almost always
- Sometimes
- Many times
- Only some of the time
- Never

For the final score of the questionnaire use the calculator available at the following link:

Free Online SF-12 Score Calculator - OrthoToolKit
