# Supplementary material for: Exercise prescription for the prevention and treatment of chronic diseases in primary care: Protocol of the RedExAP study
Source: PLoS One. 2024 Jul 5;19(7):e0302652. doi: 10.1371/journal.pone.0302652 (PMC11226089; doi:10.1371/journal.pone.0302652)
Supplement: Attachment — Submitted filename: Comments to the authors.docx [file pone.0302652.s002.docx]

Comments to the authors

I would like to congratulate the authors on a well compiled piece of work, including the basic steps of generating a guideline, which I find to be very relevant to patients, health workers and society in general, not to forget the economy behind treating chronic diseases.

I find the protocol well written, clear and concise.

I do however find some the references to be a bit “old”, and there are more recent studies on the subject than those included in the study. This is not a fault as such, but hopefully some of the newer studies will be found in the future systematic review intended as part of the upcoming study.

I have as such no comments to the authors, except I hope all the best for the coming projects. There is a large amount of work ahead, and I hope to see the result in time forthcoming.
