# Supplementary material for: Exercise prescription for the prevention and treatment of chronic diseases in primary care: Protocol of the RedExAP study
Source: PLoS One. 2024 Jul 5;19(7):e0302652. doi: 10.1371/journal.pone.0302652 (PMC11226089; doi:10.1371/journal.pone.0302652)
Supplement: Attachment — Submitted filename: PONE-D-23-38977_Review_Jan2024.docx [file pone.0302652.s003.docx]

Title:

PONE-D-23-38977: Exercise research for the prevention and treatment of chronic diseases in primary care: protocol for the rationale of the RedExAP study.

General comments:

Thank you for giving me the opportunity to review this manuscript. It is an interesting manuscript describing a research protocol consisting of two phases of study i.e. a qualitative study and clinical practice guidelines (CPG) development. However, further details are required to add clarity to the manuscript.

Specific comments:

Title:

1. ‘Exercise research’ is rather vague and can be improved to reflect more specifically what this exercise research is about.

Introduction:

2. The first and second paragraphs describe the benefits / effectiveness of exercise versus pharmacological intervention. However, it is not stated what drugs / what condition or disease(s) were previously studied. This is in consideration that in many diseases, medications have specific function in treatment. It may be difficult to compare head-to-head specific medication with exercise especially looking at mortality outcome.

3. The third paragraph: ‘….physical exercise has proven to be superior to the pharmacological treatments commonly used in clinical practice in some cases…’ Suggest to provide examples of diseases to explain ‘some cases’.

4. The term ‘rehabilitator’ – does it mean rehabilitation medicine physicians only or includes other rehabilitation therapist?

5. This sentence need reference: ‘….health services have incorporated the prescription of physical activity into the range of services they offer, some evidence shows that either this service does not reach users or is provided inadequately.’

6. The fifth paragraph describes ‘the urgency for this evidenced scientific knowledge to be accessible to healthcare professionals.’ Suggest adding references to support the statement that knowledge on exercise is not accessible.

7. Aim number (iii) is not clear. The aim is to synthesize evidence on the effectiveness of physical exercise prescription. This is more appropriately conducted through a systematic review.

Material and methods:

8. First line – qualitative study is to explore rather than analyse the barriers and facilitators.

Will the results obtained in the qualitative study contribute to the development of the CPG?

*Qualitative study*

9. Need to include operative definitions of terms used in this research: ‘primary care professionals’, ‘patients with chronic conditions’, ‘primary care centres.’

10. The description of ‘criteria’ for selecting primary care professionals or patients is confusing. Does it mean the inclusion criteria or the different groups for intentional (purposive) sampling? Therefore, need to be clarified.

11. What are the criteria to decide a participant to be of low, medium or high socioeconomic level?

12. Suggest including a short description of SF-12 scale or include it as an appendix.

13. A short description of how the topic guideline is developed should be included.

14. The regions or location of the health/primary care centres involved in participant recruitment should be included. Are they rural or urban areas?

15. ‘Data triangulation by three investigators’ need to be clarified – how will it be done?

*CPG development*

16. Refer to comment (7) regarding the appropriate method of synthesizing scientific evidence on effectiveness of physical exercise prescription. The general aim of CPG development is to provide an evidence-based guide/recommendations for health care providers to improve quality of health care. The authors need to clarify the aims. ‘Most prevalent chronic conditions’ need to be defined.

17. Point (1) following the first paragraph is confusing. Does it mean that healthcare professionals and patients will be the expert panel in the CPG development? What is meant by ‘all individuals need to declare their interests?’

18. Table 1 – the outcomes in this table are different from what is stated in the paragraph below the table 1 i.e. quality of life etc.

19. The generic question of ‘is health education on physical activity/exercises provided by professionals’ need clarification as it does not seem to align with the CPG’s aims.

20. The sentence ‘The application of evidence-based medicine is responsible for the current changes….’ Is not clear and would benefit from a re-structuring of the sentence.

Discussion:

21. Need a definition for ‘physical educators.’

22. It is stated that this research ‘…will highlight new social needs…’ however, this is not one of the study’s aim.

23. The second paragraph on economic of physical inactivity is more relevant as part of introduction to the study.

24. Page 21 – need reference to support the statement regarding health crisis due to lack of healthcare personnel. Furthermore, it does not reflect the aim of this study.

25. ‘Social determinants of health’ need further elaboration.

26. The statement; ‘study aims to recover the health system's approach from the 1980s when health promotion was an essential part of the primary care strategy’ needs clarification. Is it a specific region’s health system approach? World Health Organization still highlights health promotion as a component of primary health care (not just in the 1980s). Refer to: <https://www.who.int/health-topics/primary-health-care#tab=tab_1>
