# Supplementary material for: Exercise prescription for the prevention and treatment of chronic diseases in primary care: Protocol of the RedExAP study
Source: PLoS One. 2024 Jul 5;19(7):e0302652. doi: 10.1371/journal.pone.0302652 (PMC11226089; doi:10.1371/journal.pone.0302652)
Supplement: Attachment — Submitted filename: PONE-D-23-38977_Review_March2024.docx [file pone.0302652.s005.docx]

Title:

PONE-D-23-38977-R1: Effect of exercise for the prevention and treatment of chronic diseases in primary care: rationale of the RedExAP study.

General comments:

The authors have addressed the previous comments. However, there are a few further comments and clarifications required as below.

Specific comments:

Title:

The aims of this study are to explore barriers and facilitators for physical exercise prescription and developing a CPG for effectiveness of physical exercise prescription (and not to determine the effect of exercise). The authors may consider the title as ‘Exercise prescription for the prevention and treatment of chronic diseases.…., instead of ‘effect of exercise’ as more appropriate in reflecting the study.

Material and methods:

*Qualitative study*

In the paragraph on inclusion criteria, the authors described the socio-demographic information of the respondents that is planned to be collected and how it is to be categorized (which is also important in the methods section). Inclusion criteria by definition is which type of population can participate in the study.

Page 45, first paragraph – a topic guide would include questions to ask the participants during the focus group discussion (as a guide for the focus group moderator). To ask the participants regarding the diseases listed does not seem to align with the aim of exploring barriers and facilitators for physical exercise prescription.

Page 45 – ‘A minimum of two focus groups will be conducted per segment’, it is not clear what constitutes ‘segment’.

Page 45 – ‘Data collection and analysis will follow an interactive circular process’ – the word ‘interactive’ should be ‘iterative’.

Discussion:

Not all readers are familiar with 2030 Agenda, therefore, the authors can be more specific on this agenda. Similarly, 2030 Agenda is stated in the abstract.

Others:

‘Diabetes mellitus’ should be in lower case.

‘CINHAL’ – the correct spelling is CINAHL.

There are missing information in the revised main manuscript as below:

i. Ethical consideration

ii. Funding details

iii. Ethics approval and consent to participate.
