# Supplementary material for: Exercise prescription for the prevention and treatment of chronic diseases in primary care: Protocol of the RedExAP study
Source: PLoS One. 2024 Jul 5;19(7):e0302652. doi: 10.1371/journal.pone.0302652 (PMC11226089; doi:10.1371/journal.pone.0302652)
Supplement: Attachment — Submitted filename: PONE-D-23-38977_Review_March2024.docx [file pone.0302652.s006.docx]

Additional Editor Comments:

==============================
**ACADEMIC EDITOR:**

Comments from PLOS editorial office: When you resubmit, please modify the title of your submission to include the words "study protocol".

**Authors:**

Thank you for the reviewer’s comment. We greatly appreciate the reviewer´s time in reviewing the manuscript. As suggested, we have modified the title of the manuscript as follows:

*“Exercise prescription* for the prevention and treatment of chronic diseases in primary care: *protocol* of the RedExAP study.”

**REVIEWER 2:**

Title:

PONE-D-23-38977-R1: Effect of exercise for the prevention and treatment of chronic diseases in primary care: rationale of the RedExAP study.

General comments:

The authors have addressed the previous comments. However, there are a few further comments and clarifications required as below.

**Authors:**

Thank you for the reviewer’s comment. We greatly appreciate the reviewer´s time in reviewing the manuscript.

Specific comments:

Title:

The aims of this study are to explore barriers and facilitators for physical exercise prescription and developing a CPG for effectiveness of physical exercise prescription (and not to determine the effect of exercise). The authors may consider the title as ‘Exercise prescription for the prevention and treatment of chronic diseases.…., instead of ‘effect of exercise’ as more appropriate in reflecting the study.

**Authors:**

Thank you for the reviewer’s comment. As suggested, we have modified the title of the manuscript as follows:

*“Exercise prescription* for the prevention and treatment of chronic diseases in primary care: *protocol* of the RedExAP study.”

Material and methods:

*Qualitative study*

In the paragraph on inclusion criteria, the authors described the socio-demographic information of the respondents that is planned to be collected and how it is to be categorized (which is also important in the methods section). Inclusion criteria by definition is which type of population can participate in the study.

**Authors:**

The reviewer´s comment seems judicious. As suggested, we have included the population type.

“To organize the primary care professionals' focus groups, the following inclusion criteria will be employed: *adult subjects (over 18 years of age),* gender (male, female), area of expertise (medicine, nursing, physiotherapy), and years of professional experience (<10, 10-20, >20). For the selection of patients with chronic conditions, the following inclusion criteria will be used: *healthy adult subjects or patients with chronic conditions (over 18 years of age),* gender (male, female), employment status (retired, employed, unemployed, student), socioeconomic level (a socioeconomic level index was calculated with the items that referred to education level and occupation, classifying the socioeconomic level as: low, medium low, medium, medium high and high according to the scale proposed by the Spanish Society of Epidemiology) [26], education level (higher education or intermediate degree, high school graduate, or no education), and physical status (assessed using the SF-12 scale) (Appendix 1).”

Page 45, first paragraph – a topic guide would include questions to ask the participants during the focus group discussion (as a guide for the focus group moderator). To ask the participants regarding the diseases listed does not seem to align with the aim of exploring barriers and facilitators for physical exercise prescription.

**Authors:**

Thank you for the reviewer’s comment. As suggested, we have explained this issue as follows:

“[…] The areas included in the topic guidelines were the most prevalent chronic diseases in Primary Care (ischemic heart disease, Alzheimer's disease and other dementias, cerebrovascular accident, cancer, chronic obstructive pulmonary disease, kidney disease, hypertension and diabetes mellitus) [27] *to explore the barriers and facilitators to physical exercise prescription by primary care professionals in patients with chronic pathologies, and to explore patient barriers and facilitators and how these chronic pathologies influence compliance with physical exercise prescription.* […]”

Page 45 – ‘A minimum of two focus groups will be conducted per segment’, it is not clear what constitutes ‘segment’.

**Authors:**

The reviewer´s comment seems judicious. As suggested, we have modified this term and have explained it.

“A minimum of two focus groups will be conducted per *group, i.e. for primary care professionals and for patients,* but the final number will be determined by the data saturation criterion, which is reached when no novel analytical information is obtained [28].”

Page 45 – ‘Data collection and analysis will follow an interactive circular process’ – the word ‘interactive’ should be ‘iterative’.

**Authors:**

Thank you for the reviewer’s comment. As suggested, we have replaced the word "interactive" with "iterative".

“Data collection and analysis will follow an *iterative* circular process, where data collected in each focus group will refine the topic guidelines for subsequent groups. All data will be compared using the constant comparison method.”

Discussion:

Not all readers are familiar with 2030 Agenda, therefore, the authors can be more specific on this agenda. Similarly, 2030 Agenda is stated in the abstract.

**Authors:**

The reviewer´s comment seems judicious. As suggested, we have specified the main aims of 2020 Agenda and their relationship to physical exercise.

In the Abstract section:

“[…] The innovative transdisciplinary approach aligns with the 2030 Agenda, *promoting better population health and greater social well-being,* and shows promise in alleviating chronic disease burdens. […]”

In the Discussion section:

“[…] These changes would result in better population health and greater social well-being, a key goal of the 2030 Agenda, *which is based on the principles of sustainability, equity, inclusion and respect for human rights, promoting health and the well-being of the population where the promotion of physical exercise is a fundamental support* [44]. […]”

Others:

‘Diabetes mellitus’ should be in lower case.

**Authors:**

Thank you for the reviewer’s comment. As suggested, we have included the term “diabetes mellitus” in lower case throughout the manuscript.

“In recent years, numerous published studies have assessed the effectiveness of physical exercise in the preventive or therapeutic management of many prevalent chronic conditions that affect children [5,6], adults and the elderly [7], and, although the evidence is limited, physical exercise has proven to be superior or potentially similar to the pharmacological treatments commonly used in clinical practice in some cases, such as in patients with stroke, heart failure or *diabetes mellitus*, among other [2]; […]”

“[…] The areas included in the topic guidelines were the most prevalent chronic diseases in Primary Care (ischemic heart disease, Alzheimer's disease and other dementias, cerebrovascular accident, cancer, chronic obstructive pulmonary disease, kidney disease, hypertension and *diabetes mellitus*) [27]. […]”

‘CINHAL’ – the correct spelling is CINAHL.

**Authors:**

Thank you for the reviewer’s comment. As suggested, we have modified the word CINHAL to CINAHL.

“The most appropriate databases (such as SPORTDiscus, Medline, *CINAHL*, and Embase) were selected.”

There are missing information in the revised main manuscript as below:

i. Ethical consideration

ii. Funding details

iii. Ethics approval and consent to participate.

**Authors:**

Thank you for the reviewer’s comment. Information on ethical consideration, funding details, and ethics approval and consent to participate are shown in the unblinded main manuscript.
